# Economic burdens of health expenditure for multi-morbidity of older people with hypertension in China and Vietnam

**DOI:** 10.3389/fpubh.2025.1666119

**Published:** 2025-09-26

**Authors:** Nguyen Khanh Phuong, Nguyen Hoang Giang, Haolin Li, Nguyen The Vinh, Tran Thi Mai Oanh, Chenkai Wu

**Affiliations:** ^1^Health Strategy and Policy Institute, Hanoi, Vietnam; ^2^Global Health Research Center, Duke Kunshan University, Kunshan, China

**Keywords:** hypertension, multimorbidity, financial burden, older adults, low- and middle-income countries

## Abstract

**Background:**

Hypertension is prevalent in older adults and often coexists with other chronic diseases, increasing healthcare costs, especially in low- and middle-income countries (LMICs) like China and Viet Nam. These nations are experiencing rapid population aging, yet comparative evidence on the economic burden of hypertension-related multimorbidity is lacking.

**Objective(s):**

This study aimed to compare the financial burden, measured by out-of-pocket health expenditures (OOPHE) and catastrophic health expenditures (CHE), associated with hypertension and common comorbidities (joint disease, heart disease, and diabetes) among older adults in China and Viet Nam.

**Methods:**

Researchers analyzed data from national surveys in China (2018) and Viet Nam (2020) for adults aged 60+. They categorized hypertensive individuals by comorbidities and used regression models to assess the link between these disease clusters and OOPHE and CHE, adjusting for socioeconomic factors. CHE was defined as health spending at or above 40% of a household's capacity to pay.

**Findings:**

Comorbidities were common in both countries. In China, CHE prevalence was highest for individuals with hypertension and heart disease (30.8%), which was the comorbidity posing the greatest financial risk. In Viet Nam, the hypertension-diabetes cluster had the highest CHE risk (13.5%). In China, all comorbidity groups were associated with higher OOPHE. In Viet Nam, however, only the hypertension-diabetes group showed a significant increase in OOPHE.

**Conclusions:**

Multimorbidity significantly increases the financial burden for older adults with hypertension, with the key impactful diseases varying by country. Heart disease imposes the greatest burden in China, while diabetes is the primary driver in Viet Nam. Targeted policies, such as improving outpatient coverage and tailoring benefits to common disease combinations, are crucial to protect aging populations in LMICs from financial hardship.

## Introduction

Hypertension has become a major global public health challenge in recent decades, affecting over 1 billion individuals worldwide, with a disproportionate burden in low- and middle-income countries (LMICs) (1). This condition frequently clusters with other chronic conditions such as joint diseases, heart diseases, and diabetes, which collectively complicates clinical care and exacerbates health risks (2). Older adults are particularly affected by these co-occuring conditions, facing heightened risks of disability and financial hardship due to sustained healthcare demands (3).

The economic implications of multi-morbidity are profound, particularly in resource-constrained environments. Previous research has demonstrated that multi-morbidity exacerbated out-of-pocket (OOP) health expenditures and catastrophic health expenditures (CHE), placing significant financial strain on affected households (4). In LMICs, the management of concurrent chronic conditions escalates healthcare costs and intensifies economic risks, with older adults often experiencing severe financial distress (5).

China and Vietnam are experiencing rapid population aging. By 2040, it is projected that 28% of China's population will be aged 60 and over (6). Studies in China indicated a rising incidence of CHE with multimorbidity. In a longitudinal study, Chinese older adults with any chronic disease had a 30% higher risk of CHE than those with none (7). Another study found that 36.7% of widowed older adults had experienced CHE, with the highest risk among those in “cardiovascular” or “multisystem” multimorbidity clusters (8). In Vietnam, older adults constituted 11.9% of the total population in 2019, and this figure is projected to surpass 25% by 2050 (9). Studies on multimorbidity and CHE in Vietnam are scarce. However, surveys of older households highlighted high OOP burden. One study reported that spending by older members made up 86.3% of total household health costs, and 8.6% of households with seniors faced CHE (10). Financially, Vietnam has expanded social health insurance, which has improved access and protected many older citizens (11). However, studies show vulnerable older adults (rural, ethnic minorities, or lower-income) still incur high catastrophic spending despite insurance (10, 12). Vietnam's fast-aging population is stressing public finances: ensuring equitable coverage and affordable care for multimorbid elders is a key challenge.

Despite these insights, comparative research on the economic impact of multimorbidity among hypertensive older adults in LMICs is lacking. This gap impedes targeted policy development to reduce financial hardship in aging populations with complex health needs. To address this, we examined the medical economic burden using data from China and Vietnam—two nations where aging populations and evolving disease profiles intersect with distinct health financing frameworks. China, classified as an upper-middle-income country, has been expanding its social health insurance system, whereas Vietnam, a lower-middle-income country, continues to rely predominantly on OOP payments (13).

This study aimed to quantify and compare OOP expenditures and CHE among older adults with hypertension alone and those with hypertension accompanied by at least one common co-occurring chronic condition (joint diseases, heart diseases, or diabetes) in China and Vietnam. By highlighting financial risk disparities in these two middle-income countries, the findings intend to inform context-specific strategies for better financial protection of vulnerable populations facing multimorbidity.

## Methods

### Study design and data sources

This study was a secondary data analysis utilizing two population-based household surveys conducted in China and Vietnam.

In China, we used data from the 2018 wave of the China Health and Retirement Longitudinal Study (CHARLS), a nationally representative longitudinal survey enrolled residents aged 45 years and older. To enhance comparability with the Vietnam dataset, three provinces (Sichuan, Jiangsu, and Yunnan) were selected based on their GDP levels and geographic distribution. CHARLS employed a multistage stratified probability-proportional-to-size sampling design, with data collected through face-to-face interviews using standardized structured questionnaires. The survey also included physical measurements and biomarker data. To ensure national representativeness, individual weighting variables were applied. Detailed information on CHARLS design and implementation has been published elsewhere (14). For this study, we extracted data for individuals aged 60 years and above, yielding a sub-sample of 1,358 older adults. Among these, 625 individuals were identified as having hypertension, based on self-reports, blood pressure measurements, or current use of antihypertensive medications (15) (Supplementary Table S1). These individuals belonged to 523 unique households included in our analysis.

In Vietnam, we used data from a cross-sectional household survey conducted in 2020 across three provinces representing the northern (Yen Bai), central (Thanh Hoa), and southern (Tien Giang) regions of the country. The survey employed a multistage cluster sampling design to recruit a representative sample of 1,536 individuals aged 60 years and older. The study employed multi-stage sampling by first stratifying districts into urban and rural areas and randomly selecting one from each. Two communes were then chosen from each selected district to form 12 clusters. Within each cluster, residents aged 60 and over were stratified by age (60–69, 70–79, and ≥80 years), from which a final sample of 128 individuals per commune was drawn using proportionate random sampling to ensure the sample's age structure reflected that of the commune's population. The methodology and sampling approaches of the survey have been described in detail elsewhere (10) (Supplementary Table S1). For this study, we focused on a sub-sample of 734 older individuals who self-reported a diagnosis of hypertension, corresponding to 723 households.

In both countries, data were collected through structured, interviewer-administered questionnaires. The instruments included modules on individuals' health status, self-reported diagnosis of chronic conditions, healthcare utilization, and associated out-of-pocket expenditures. Additionally, data on household-level characteristics such as demographic composition, income, consumption were collected. These comprehensive datasets provide an opportunity to analyze household-level economic impacts of NCD multi-morbidity among older adults with hypertension in two contrasting health system and socioeconomic contexts.

### Comorbidity

In this study, NCD comorbidity status refers to older adults diagnosed with hypertension who also suffer from at least one additional chronic condition, namely joint diseases, heart diseases, or diabetes. These three comorbidity categories were selected due to their high prevalence among the older individuals in our study sample (Supplementary Table S2). A household was classified as having an older person with NCD comorbidity if any older resident met the criteria mentioned above. In contrast, a household was categorized as having hypertension only if at least one older member had hypertension without any of the additional chronic conditions, and all other older members in the household were free from chronic conditions.

### Outcomes

This study analyzed the economic burden of health expenditures on older adults at both the individual and household levels. At the individual level, we estimated the out-of-pocket health expenditure (OOPHE) specifically for older adults within the household.

In China, the household OOPHE was defined as the combined out-of-pocket spending by older people and their spouses for outpatient and inpatient care over the past year. In Vietnam, household OOPHE was defined as the direct spending on healthcare by older people and other household members (children and others), including expenditures on outpatient and inpatient services, home-based care for older adults, self-medication, and the purchase of medical devices. All OOPHE values were annualized to reflect 12-month expenditures. OOPHE was treated as a continuous variable reflecting the monetary value of health expenditures.

To assess the financial burden of health expenditures at the household level, we identified households as incurring catastrophic health expenditures (CHE) if their out-of-pocket health spending equaled or exceeded 40% of their capacity to pay, following the WHO's methodology (16). The household's capacity to pay was calculated as total household expenditure minus food expenditure. A binary variable was constructed to indicate whether a household incurred CHE (1 = yes; 0 = no).

### Covariates

Covariates were selected based on relevant literature and data availability. Individual-level characteristics included age, sex (male or female), insurance status (insured vs. uninsured), household size, and household living standards. To assess living standards in Vietnam, a wealth index was constructed using principal component analysis (17), incorporating variables such as household assets, durable goods, housing construction materials, sources of drinking water, sanitation facilities, and agricultural land ownership. The analysis also accounted for differences in asset ownership and amenities patterns between urban and rural areas (18). Based on the resulting wealth index, households were grouped into five wealth quintiles (Q1–Q5), with Q1 representing the poorest 20% and Q5 representing the wealthiest 20%. For Chinese households, total household income per capita was used to classify households into comparable wealth quintiles.

### Statistical analysis

Separate analyses were conducted for the datasets from China and Vietnam. Results were presented by the three NCD comorbidity categories, alongside comparison to the reference group of older adults with hypertension alone. Descriptive statistics were first employed to summarize the characteristics of older individuals and their households. This also examined the differences in positive OOPHE—which encompassed costs such as outpatient care, inpatient care, self-medication, and more (if any)—and the occurrence of CHE due to these expenses. Next, multivariate logistic regression models were used to assess the association between NCD comorbidity and the likelihood of incurring CHE, while two-part models were applied for the analysis of OOPHE level. The two-part models were used due to the skewed distribution of the dependent variable (i.e., OOPHE), which contained a massive number of zero-expenditure observations. In the first part, logistic regression was used to estimate the probability of incurring positive OOPHE in the last 12 months, comparing individuals in each comorbidity group to those with hypertension only (reference group). In the second part, for those who reported positive OOPHE, a generalized linear model with a gamma distribution and a log link function was used to estimate the expected amount of OOPHE. All regression models were adjusted using individual-level sampling weights and controlled for the individual-level characteristics, including age, sex, insurance status, household wealth quintile, and household size.

### Ethical clearance

This study involved secondary analysis of de-identified data from two previously conducted population-based surveys in China and Vietnam. In China, the original data were obtained from the CHARLS, which received ethical approval from the Biomedical Ethics Review Committee of Peking University (approval number: IRB00001052-11015). All participants in the CHARLS study provided written informed consent prior to data collection.

In Vietnam, the original household survey was conducted by the Health Strategy and Policy Institute (HSPI) and received ethical approval from the Institutional Scientific Research Committee of HSPI, Hanoi. Ethical clearance was also granted by the WHO Research Ethics Review Committee (protocol number: ERC.0003085). Written informed consent was obtained from all study participants before participation. The current secondary data analysis was conducted using anonymized datasets with no personal identifiers. The study protocol for this analysis was reviewed and approved by the Institutional Scientific Research Committee of the HSPI, which confirmed that the use of these secondary data complied with ethical research standards and relevant data sharing agreements. No additional ethical risks were identified.

## Results

### Sample description

Among the 1,358 Chinese participants, 46.0% had hypertension, commonly comorbid with joint disease (22.5%), heart disease (16.2%), and diabetes (10.6%; Supplementary Table S2). Compared to those with hypertension only, individuals with at least hypertension and joint disease were more likely to be female (56.8 vs. 44.3%), reside in rural areas (66.0 vs. 48.6%), be insured (97.1 vs. 95.7%), and be illiterate (43.5 vs. 35.7%; Table 1). Those with at least hypertension and heart disease were older (43.0% aged 70–79 vs. 37.1%), more often female (54.2 vs. 44.3%), more likely to reside in rural areas (55.6 vs. 48.6%), and had greater insurance coverage (98.6 vs. 95.7%). Participants with at least hypertension and diabetes also tended to be older (36.1% aged 70–79 vs. 37.1%), more likely female (50.8 vs. 44.3%), rural residents (48.4 vs. 51.4%), married (79.5 vs. 75.7%), and insured (95.9 vs. 95.7%). In terms of household level comparison to households containing only hypertensive individuals, those with hypertension and comorbidities had fewer single-older-adult households (9.7–12% vs. 33.3%) and were more often urban (around 34.4–47.8% vs. 53.3%). Households with hypertension and diabetes showed higher wealth (31% richest quintile vs. 30%; Table 2).

**Table 1:** Background characteristics of older people with NCD comorbidities in China.

	**Participants with at least hypertension+joint (*n* = 377)**	**Participants with at least hypertension+heart (*n* = 144)**	**Participants with at least hypertension+diabetes (*n* = 122)**	**Hypertension only (*n* = 70)**	**No chronic conditions (*n* = 151)**	**Total (*n* = 1,358)**
**Age in groups**, ***n*** **(%)**						
60–69	200 (53.0%)	60 (41.7%)	63 (51.6%)	35 (50.0%)	99 (65.6%)	770 (56.7%)
70–79	127 (33.7%)	62 (43.0%)	44 (36.1%)	26 (37.1%)	33 (21.8%)	436 (32.1%)
80+	50 (13.3%)	22 (15.3%)	15 (12.3%)	9 (12.9%)	19 (12.6%)	152 (11.2%)
**Sex**, ***n*** **(%)**						
Male	163 (43.2%)	66 (45.8%)	60 (49.2%)	39 (55.7%)	87 (57.6%)	670 (49.3%)
Female	214 (56.8%)	78 (54.2%)	62 (50.8%)	31 (44.3%)	64 (42.4%)	688 (50.7%)
**Place of residence**, ***n*** **(%)**						
Urban	128 (34.0%)	64 (44.4%)	59 (48.4%)	36 (51.4%)	70 (46.4%)	554 (40.8%)
Rural	249 (66.0%)	80 (55.6%)	63 (51.6%)	34 (48.6%)	81 (53.6%)	804 (59.2%)
**Marital status**, ***n*** **(%)**						
Married	259 (68.7%)	106 (73.6%)	97 (79.5%)	53 (75.7%)	123 (81.5%)	1,038 (76.4%)
Other	118 (31.3%)	38 (26.4%)	25 (20.5%)	17 (24.3%)	28 (18.5%)	320 (23.6%)
**Insurance coverage**, ***n*** **(%)**						
Yes	366 (97.1%)	142 (98.6%)	117 (95.9%)	67 (95.7%)	151	1,321 (97.3%)
No	11 (2.9%)	2 (1.4%)	5 (4.1%)	3 (4.3%)	0	37 (2.7%)
**Education level**, ***n*** **(%)**						
Illiterate	164 (43.5%)	52 (36.1%)	44 (36.0%)	25 (35.7%)	61 (40.4%)	516 (38.0%)
Below elementary school	94 (24.9%)	34 (23.6%)	24 (19.7%)	13 (18.6%)	25 (16.6%)	317 (23.4%)
Elementary school	91 (24.2%)	37 (25.7%)	29 (23.8%)	22 (31.4%)	33 (21.8%)	329 (24.2%)
Middle school and above	28 (7.4%)	21 (14.6%)	25 (20.5%)	10 (14.3%)	32 (21.2%)	196 (14.4%)

**Table 2:** Background of households having people with NCD comorbidities in China.

	**Hypertension+joint (*n* = 343)**	**Hypertension+heart (*n* = 134)**	**Hypertension+diabetes (*n* = 113)**	**Hypertension (*n* = 30)**	**No chronic condition (*n* = 51)**	**Total (*n* = 864)**
Urban households, *n* (%)	118 (34.4%)	59 (44.0%)	54 (47.8%)	16 (53.3%)	22 (43.1%)	344 (39.8%)
**No. of people in households (**≥**60)**						
1	41 (12.0%)	14 (10.4%)	11 (9.7%)	10 (33.3%)	13 (25.5%)	130 (15.0%)
2	180 (52.5%)	81 (60.5%)	70 (61.9%)	14 (46.7%)	22 (43.1%)	442 (51.2%)
3	41 (11.9%)	13 (9.7%)	10 (8.9%)	3 (10.1%)	6 (11.8%)	110 (12.7%)
4	28 (8.1%)	9 (6.7%)	10 (8.9%)	1 (3.3%)	6 (11.8%)	76 (8.8%)
5	35 (10.2%)	12 (9.0%)	6 (5.3%)	1 (3.3%)	2 (3.9%)	65 (7.5%)
6+	18 (5.3%)	5 (3.7%)	6 (5.3%)	1 (3.3%)	2 (3.9%)	41 (4.8%)
Household with children co-reside	133 (38.8%)	43 (32.1%)	36 (31.9%)	10 (33.3%)	22 (43.1%)	348 (40.3%)
**No. of people aged** ≥**60 years in the household**						
1 person	41 (12.0%)	14 (10.4%)	11 (9.7%)	10 (33.3%)	13 (25.5%)	130 (15.0%)
≥2 persons	302 (88.0%)	120 (89.6%)	102 (90.3%)	20 (66.7%)	38 (74.5%)	734 (85.0%)
**Household wealth quintile (using total household income per capita)**						
Q1 (Poorest)	80 (23.3%)	22 (16.4%)	18 (15.9%)	8 (26.7%)	14 (27.4%)	181 (21.0%)
Q2	68 (19.9%)	22 (16.4%)	20 (17.7%)	8 (26.7%)	9 (17.7%)	173 (20.0%)
Q3	79 (23.0%)	31 (23.1%)	19 (16.8%)	3 (10.0%)	9 (17.7%)	173 (20.0%)
Q4	60 (17.5%)	25 (18.7%)	21 (18.6%)	2 (6.6%)	10 (19.5%)	170 (19.7%)
Q5 (Richest)	56 (16.3%)	34 (25.4%)	35 (31.0%)	9 (30.0%)	9 (17.7%)	167 (19.3%)

Among the 1,536 Vietnamese participants, 47.8% had hypertension, with frequent comorbidity involving joint disease (16.1%), heart disease (10.5%), and diabetes (8.5%; Supplementary Table S2). Compared to the hypertension-only group, individuals with at least hypertension and joint disease were more likely to be female (68.2 vs. 42.0%), reside in rural areas (52.6 vs. 50.4%), be insured (99.3 vs. 97.0%), and have lower education, including a higher proportion below elementary school (25.7 vs. 22.2%) and illiteracy (9.3 vs. 1.3%; Table 3). The hypertension and heart group had more participants aged 80+ (22.8 vs. 17.3%), more females (74.5 vs. 42.0%), greater rural residence (63.1 vs. 50.4%), and higher illiteracy (8.3 vs. 1.3%). In the hypertension and diabetes group, a higher proportion were aged 70–79 (37.9 vs. 21.9%), female (60.5 vs. 42.0%), urban residents (51.1 vs. 49.6%), and insured (100 vs. 97.0%). Households with hypertension and comorbidities were less likely to consist of a single older adult (10–12% vs. 36%) compared to hypertension-only households. Urban residence was lower in hypertension and joint households (39.5 vs. 54%; Table 4).

**Table 3:** Background characteristics of older people with NCD comorbidities in Viet Nam (*individual level sampling weight applied*).

	**Participants with at least hypertension+joint (*n* = 247)**	**Participants with at least hypertension+heart (*n* = 161)**	**Participants with at least hypertension+diabetes (*n* = 131)**	**Hypertension only (*n* = 195)**	**No chronic conditions (*n* = 344)**	**Total (*n* = 1,536)**
**Age in groups**, ***n*** **(%)**						
60–69	104 (47.5%)	63 (39.3%)	64 (49.2%)	96 (60.8%)	205 (65.6%)	792 (57.6%)
70–79	102 (34.8%)	65 (37.9%)	48 (37.9%)	65 (21.9%)	101 (23.0%)	515 (26.8%)
80+	41 (17.7%)	33 (22.8%)	18 (12.9%)	34 (17.3%)	38 (11.4%)	227 (15.6%)
**Sex**, ***n*** **(%)**						
Male	73 (31.8%)	48 (25.5%)	48 (39.5%)	90 (58.0%)	156 (46.2%)	632 (43.7%)
Female	174 (68.2%)	113 (74.5%)	83 (60.5%)	105 (42.0%)	188 (53.8%)	904 (56.3%)
**Place of residence**, ***n*** **(%)**						
Urban	129 (47.4%)	83 (36.9%)	78 (51.1%)	118 (49.6%)	144 (36.3%)	768 (43.8%)
Rural	118 (52.6%)	78 (63.1%)	53 (48.9%)	77 (50.4%)	200 (63.7%)	768 (56.2%)
**Marital status**, ***n*** **(%)**						
Married	137 (58.8%)	75 (46.0%)	74 (57.4%)	111 (62.0%)	216 (68.4%)	926 (64.6%)
Other	110 (41.2%)	86 (54.0%)	57 (42.6%)	84 (38.0%)	128 (31.6%)	610 (35.4%)
**Insurance coverage**, ***n*** **(%)**						
Yes	243 (99.3%)	157 (98.3%)	131 (100%)	190 (97.0%)	327 (95.5%)	1,481 (96.6%)
No	4 (0.7%)	4 (1.7%)	0 (0.0%)	5 (3.0%)	17 (4.5%)	55 (3.4%)
**Education level**, ***n*** **(%)**						
Illiterate	16 (9.3%)	14 (8.3%)	12 (10.3%)	2 (1.3%)	7 (2.8%)	51 (4.2%)
Below elementary school	56 (25.7%)	52 (35.3%)	33 (30.9%)	34 (22.2%)	63 (22.6%)	305 (22.8%)
Elementary school	41 (14.1%)	31 (16.3%)	18 (10.0%)	41 (20.3%)	60 (18.9%)	304 (19.5%)
Middle school and above	134 (50.9%)	64 (40.1%)	68 (48.8%)	118 (56.2%)	214 (55.7%)	875 (53.5%)

**Table 4:** Background of households having people with NCD comorbidities in Viet Nam (*household level sampling weight applied*).

	**Hypertension+joint (*n* = 244)**	**Hypertension+heart (*n* = 159)**	**Hypertension+diabetes (*n* = 130)**	**Hypertension only (*n* = 190)**	**No chronic condition (*n* = 327)**	**Total (*n* = 1,477)**
Urban households, *n* (%)	127 (47.5%)	80 (36.1%)	77 (51.1%)	115 (50.4%)	134 (36.3%)	720 (42.6%)
**No. of people in households**						
1	33 (13.8%)	27 (15.9%)	13 (7.0%)	24 (8.9%)	49 (12.2%)	191 (11.1%)
2	97 (40.0%)	52 (30.3%)	47 (30.4%)	64 (31.3%)	124 (33.4%)	549 (36.0%)
3	27 (8.2%)	19 (12.1%)	14 (13.1%)	26 (16.3%)	45 (13.1%)	179 (11.2%)
4	22 (8.7%)	20 (16.8%)	13 (10.8%)	21 (13.1%)	31 (11.0%)	162 (11.8%)
5	33 (16.3%)	15 (10.3%)	19 (19.1%)	26 (15.5%)	34 (12.9%)	181 (14.0%)
6+	32 (13.0%)	26 (14.6%)	24 (19.6%)	29 (14.8%)	44 (17.5%)	215 (15.9%)
Household with children co-reside	40 (19.3%)	18 (16.4%)	19 (24.0%)	29 (17.0%)	49 (18.1%)	231 (18.4%)
**No. of people aged** ≥**60 years in the household**						
1 person	113 (50.9%)	83 (53.9%)	59 (49.7%)	87 (45.6%)	142 (39.6%)	642 (42.7%)
≥2 persons	131 (49.1%)	76 (46.1%)	71 (50.3%)	103 (54.4%)	185 (60.4%)	835 (57.3%)
**Household wealth quintile (using asset-based wealth index)**						
Q1 (Poorest)	40 (19.3%)	32 (21.2%)	17 (14.5%)	42 (28.7%)	76 (23.1%)	296 (22.0%)
Q2	49 (21.5%)	34 (24.3%)	25 (21.1%)	33 (17.0%)	58 (20.0%)	295 (21.7%)
Q3	58 (19.9%)	39 (21.7%)	26 (18.4%)	36 (18.9%)	64 (17.5%)	296 (16.7%)
Q4	43 (13.5%)	34 (18.7%)	31 (25.4%)	37 (13.9%)	65 (19.1%)	295 (17.5%)
Q5 (Richest)	54 (25.8%)	20 (14.1%)	31 (20.6%)	42 (21.5%)	64 (20.3%)	295 (22.1%)

Hypertension prevalence was similar in China (46.0%) and Vietnam (47.8%), with joint disease, heart disease, and diabetes as common comorbidities. In both countries, people with these comorbidities tended to be older, more often female, and more likely to have insurance compared to those with hypertension alone. Vietnam showed a stronger female predominance and higher rural residence among those with comorbidities, while China had notably higher illiteracy rates. At the household level, households with comorbidities in both countries were less likely to consist of a single older adult compared to those with hypertension only. Furthermore, urban residence was lower among Vietnam's hypertension and joint disease households, while urban proportions in China were more consistent across groups. Hypertension and its common comorbidities were prevalent in both China and Vietnam, showing distinct sociodemographic and household patterns.

### Out-of-pocket health expenditures and catastrophic health expenditures

In China, CHE prevalence at the household level for hypertension was 6.7% and 26.5%, 31.3%, and 25.7% for comorbid with joint disease, heart disease, and diabetes, respectively (Table 5). Compared to households with only hypertension, comorbidity households had lower proportion of not incurring any OOP health expenditure (Total OOPHE, 36.6–45.1% vs. 83.3%). Among households incurring medical expenses, those with hypertension and diabetes exhibited the highest average expenditure (Mean Total OOPHE: $2,466), followed by the hypertension and heart disease group (Mean Total OOPHE: $2,365), while the hypertension and joint disease group incurred the lowest average expenditure (Mean Total OOPHE: $2,083).

**Table 5:** Households' health expenditure per household with individuals having at least two comorbidities among the three major types in China.

	**Hypertension+joint (*n* = 343)**	**Hypertension+heart (*n* = 134)**	**Hypertension+diabetes (*n* = 113)**	**Hypertension (*n* = 30)**	**Total (*n* = 864)**
**Total OOP health expenditure (in USD)**					
$0	154 (44.9%)	49 (36.6%)	51 (45.1%)	25 (83.3%)	463 (53.6%)
Above $0–$1,000	115 (33.5%)	46 (34.3%)	34 (30.1%)	5 (16.7%)	250 (28.9%)
$1,000–$2,000	36 (10.5%)	18 (13.4%)	14 (12.4%)	0	71 (8.2%)
$2,000 and above	38 (11.1%)	21 (15.7%)	14 (12.4%)	0	80 (9.3%)
$Mean, (SD) for households with expenditure	2,083 (4,030) [*n* = 189]	2,365 (3,600) [*n* = 85]	2,466 (3,988) [*n* = 62]	203 (214) [*n* = 5]	1,889 (3,510) [*n* = 401]
$Median, (IQR) for households with expenditure	735 (1,623) [*n* = 189]	842 (1,624) [*n* = 85]	750 (1,449) [*n* = 62]	110 (218) [*n* = 5]	612 (1,471) [*n* = 401]
**OOPHE for outpatient care (in USD)**					
$0	241 (70.3%)	84 (62.7%)	83 (73.5%)	27 (90.0%)	646 (74.8%)
Above $0–$1,000	69 (20.1%)	28 (20.9%)	22 (19.5%)	3 (10.0%)	147 (17.0%)
$1,000 and above	33 (9.6%)	22 (16.4%)	8 (7.0%)	0	71 (8.2%)
$Mean, (SD) for households with expenditure	1,908 (3,638) [*n* = 102]	1,988 (2,687) [*n* = 50]	1,779 (2,995) [*n* = 30]	135 (148) [*n* = 3]	1,586 (2,873) [*n* = 218]
$Median, (IQR) for households with expenditure	505 (1,286) [*n* = 106]	919 (1,471) [*n* = 50]	459 (897) [*n* = 30]	110 (292) [*n* = 3]	505 (1,351) [*n* = 218]
**OOPHE for inpatient care (in USD)**					
$0	209 (60.9%)	69 (51.5%)	63 (55.8%)	28 (93.3%)	584 (67.6%)
Above $0–$1,000	96 (28.0%)	47 (35.1%)	32 (28.3%)	2 (6.7%)	206 (23.7%)
$1,000 and above	38 (11.1%)	18 (13.4%)	18 (15.9%)	0	74 (8.7%)
$Mean, (SD) for households with expenditure	1,584 (3,011) [*n* = 134]	1,563 (3,059) [*n* = 65]	1,990 (3,626) [*n* = 50]	306 (325) [*n* = 2]	1,471 (2,729) [*n* = 280]
$Median, (IQR) for households with expenditure	543 (1,072) [*n* = 134]	766 (873) [*n* = 65]	651 (1,195) [*n* = 50]	306 (460) [*n* = 2]	482 (934) [*n* = 280]
Household suffering from CHE, *n* (%)	91 (26.5%)	42 (31.3%)	29 (25.7%)	2 (6.7%)	195 (22.6%)

CHE, catastrophic health expenditure.

USD to CNY = 6.5270 (Currency at 31/12/2020).

In Vietnam, CHE prevalence at the household level was 5.1% for hypertension only and 12.8%, 11.0%, and 13.5% for comorbid with joint disease, heart disease, and diabetes, respectively (Table 6). Compared to households with only hypertension, comorbidity households had a lower proportion of not incurring any OOP health expenditure (Total OOPHE 9–11% vs. 23%). Among households with medical expenses, those with hypertension and diabetes had the highest average expenditure (Mean Total OOPHE: $830, VND 19,268,450), followed by the hypertension and heart disease group (Mean Total OOPHE: $703, VND 16,320,145), while the hypertension and joint disease group had the lowest average expenditure (Mean Total OOPHE: $690, VND 16,018,350).

**Table 6:** Households' health expenditure per household with individuals having at least two comorbidities among the three major types in Viet Nam (*household level sampling weight applied*).

	**Hypertension+joint (*n* = 244)**	**Hypertension+heart (*n* = 159)**	**Hypertension+diabetes (*n* = 130)**	**Hypertension only (*n* = 190)**	**Total (*n* = 1,477)**
**Total OOP health expenditure for OPs and other family members (in USD)**					
$0	28 (10.6%)	18 (10.5%)	18 (8.9%)	49 (22.8%)	363 (23.0%)
Above $0–$1,000	173 (71.5%)	115 (69.8%)	85 (63.8%)	130 (68.4%)	969 (66.6%)
$1,000–$2,000	21 (10.6%)	17 (14.5%)	13 (17.0%)	7 (4.8%)	94 (7.0%)
$2,000 and above	22 (7.3%)	9 (5.2%)	14 (10.3%)	4 (4.1%)	51 (3.4%)
$Mean, (SD) for households with expenditure	690 (1,219) [*n* = 216]	703 (1,281) [*n* = 141]	830 (956) [*n* = 112]	427 (751) [*n* = 141]	477 (831) [*n* = 1,114]
$Median, (IQR) for households with expenditure	314 (722) [*n* = 216]	352 (772) [*n* = 141]	564 (1,042) [*n* = 112]	146 (306) [*n* = 141]	186 (493) [*n* = 1,114]
**Total OOPHE for OPs only (in USD)**					
$0	35 (14.6%)	25 (14.1%)	22 (9.3%)	145 (27.4%)	450 (28.8%)
Above $0–$1,000	168 (68.8%)	110 (68.3%)	82 (63.8%)	368 (63.9%)	895 (62.0%)
$1,000– $2,000	21 (10.5%)	15 (12.4%)	13 (19.1%)	26 (4.5%)	84 (6.1%)
$2,000 and above	20 (6.1%)	9 (5.2%)	13 (7.8%)	14 (4.1%)	48 (3.1%)
$Mean, (SD) for households with expenditure	692 (1,228) [*n* = 209]	675 (1,301) [*n* = 134]	807 (947) [*n* = 108]	400 (674) [*n* = 129]	472 (832) [*n* = 1,027]
$Median, (IQR) for households with expenditure	314 (714) [*n* = 209]	299 (660) [*n* = 134]	448 (1,041) [*n* = 108]	112 (271) [*n* = 129]	185 (491) [*n* = 2,027]
**OOPHE for outpatient care for OPs (in USD)**					
$0	119 (49.7%)	79 (52.3%)	51 (37.7%)	141 (80.4%)	993 (68.9%)
Above $0– $1,000	103 (43.6%)	68 (38.6%)	64 (49.3%)	46 (18.0%)	421 (27.2%)
$1,000 and above	22 (6.7%)	12 (9.1%)	15 (13.0%)	3 (1.6%)	63 (3.9%)
$Mean, (SD) for households with expenditure	530 (959) [*n* = 125]	646 (1,144) [*n* = 80]	633 (803) [*n* = 79]	293 (459) [*n* = 49]	450 (725) [*n* = 484]
$Median, (IQR) for households with expenditure	179 (526) [*n* = 125]	199 (732) [*n* = 80]	282 (874) [*n* = 79]	95 (224) [*n* = 49]	196 (460) [*n* = 484]
**OOPHE for inpatient care for OPs (in USD)**					
$0	190 (75.8%)	122 (79.6%)	103 (69.4%)	166 (86.4%)	1,256 (83.3%)
Above $0–$1,000	49 (21.8%)	35 (19.8%)	25 (27.3%)	23 (13.0%)	210 (15.9%)
$1,000 and above	5 (2.4%)	2 (0.6%)	2 (3.3%)	1 (0.6%)	11 (0.8%)
$Mean, (SD) for households with expenditure	284 (631) [*n* = 54]	183 (651) [*n* = 37]	246 (462) [*n* = 27]	184 (267) [*n* = 24]	233 (575) [*n* = 221]
$Median, (IQR) for households with expenditure	96 (134) [*n* = 54]	68 (86) [*n* = 37]	56 (88) [*n* = 27]	78 (157) [*n* = 24]	73 (131) [*n* = 221]
**OOPHE for self-medication for OPs (in USD)**					
$Mean, (SD) for households with expenditure	375 (445) [*n* = 83]	314 (335) [*n* = 55]	361 (413) [*n* = 32]	201 (273) [*n* = 60]	305 (383) [*n* = 416]
$Median, (IQR) for households with expenditure	168 (504) [*n* = 83]	224 (258) [*n* =55]	224 (246) [*n* = 32]	112 (112) [*n* = 60]	157 (330) [*n* = 416]
**OOPHE for home-based medical devices for Ops (in USD)**					
$Mean, (SD) for households with expenditure	51 (54) [*n* = 31]	66 (113) [*n* = 19]	52 (38) [*n* = 17]	746 (1,201) [*n* = 18]	124 (425) [*n* = 126]
$Median, (IQR) for households with expenditure	37 (43) [*n* = 31]	32 (9) [*n* = 19]	41 (34) [*n* = 17]	129 (2,727) [*n* = 18]	41 (37) [*n* = 126]
**OOPHE for long-term care for OPs (in USD)**					
$Mean, (SD) for households with expenditure	1,023 (1,400) [*n* = 19]	555 (602) [*n* = 10]	706 (609) [*n* = 9]	2,240 [*n* = 1]	640 (1,081) [*n* = 42]
$Median, (IQR) for households with expenditure	724 (866) [*n* = 19]	258 (930) [*n* = 10]	336 (838) [*n* = 9]	2,240 [*n* = 1]	168 (978) [*n* = 42]
Household suffering from CHE, *n* (%)	37 (12.8%)	24 (11.0%)	17 (13.5%)	11 (5.1%)	126 (6.3%)

CHE, catastrophic health expenditure.

USD to VND = 23,215 (Currency at 31/12/2020).

China had higher CHE rates, with 8.2% for hypertension only and between 25.8 and 30.8% for comorbidities, compared to Vietnam's 5.1% for hypertension only and 11.0%−13.5% for comorbidities. In both countries, households with comorbidities had fewer cases of zero out-of-pocket health spending than those with hypertension alone. Average total out-of-pocket costs were greater in China, where households with hypertension and diabetes spent $2,466 (CNY 16,095) on average, compared to $830 (VND 19,268,450) in Vietnam. This spending pattern was consistent across both countries, with the highest costs observed in households with hypertension and diabetes, followed by those with heart disease, and then joint disease. Comorbidities, particularly diabetes, were associated with higher out-of-pocket and catastrophic health expenditures.

### Hypertension-related multimorbidity and catastrophic health expenditure

At the individual level, each additional hypertensive individual with comorbid conditions in a household was associated with a higher risk of household-level catastrophic health expenditure (CHE), with country-specific patterns. In China, the highest odds of CHE were observed among hypertensive individuals with heart disease [odds ratio (OR) = 4.32; 95% confidence interval (CI): 1.66–11.22], then joint disease (OR = 3.52; 95% CI: 1.43–8.68) and diabetes (OR = 3.31; 95% CI: 1.24–8.82), compared to hypertension alone (Tables 7, 8). In Vietnam, hypertension with diabetes had the strongest CHE association (OR = 9.35, 95% CI: 2.38–36.80), followed by joint disease (OR = 3.16, 95% CI: 1.17–8.53), while heart disease combinations were not significant. Hypertension-related multimorbidity substantially increased the risk of catastrophic health expenditure, with country-specific patterns.

**Table 7:** Logistic regression of individual level CHE in China.

**Disease status**	***N***	**Unadjusted OR (95% CI)**	***N***	Adjusted^*^OR (95% CI)
With only hypertension		Ref.		Ref.
At least with hypertension and joint disease	447	3.21 (1.41, 7.27)	447	3.17 (1.36, 7.39)
At least with hypertension and heart disease	214	3.96 (1.65, 9.48)	214	3.88 (1.52, 9.95)
At least with hypertension and diabetes	192	3.34 (1.37, 8.08)	192	3.05 (1.14, 8.16)

OR, odds ratio; 95% CI, 95% confidence interval.

^*^Adjusted for age, sex, household wealth quintile (using household income per capita), household size (number of people in the household), health insurance status.

**Table 8:** Logistic regression of individual level CHE in Vietnam (*individual level sampling weight applied*).

**Disease status**	***N***	**Unadjusted OR (95% CI)**	***N***	Adjusted^*^OR (95% CI)
With only hypertension		Ref.		Ref.
At least with hypertension and joint disease	442	2.90 (1.06, 7.92)	442	3.16 (1.17, 8.53)
At least with hypertension and heart disease	356	2.39 (0.83, 6.86)	356	2.69 (0.97, 7.41)
At least with hypertension and diabetes	326	2.92 (0.93, 9.22)	326	9.35 (2.38, 36.8)

OR, odds ratio; 95% CI, 95% confidence interval.

^*^Adjusted for age, sex, household wealth quintile (using an asset-based wealth index), household size (number of people in the household), health insurance status.

### Association between hypertension-related comorbidities and OOP health expenditures

After adjustment for age, sex, household size, wealth quintile, and insurance status, each additional individual with hypertension-related comorbidities in a household was associated with a markedly higher probability that the household would incur any OOPHE compared with households where hypertensive individuals had no comorbidities. However, the magnitude and statistical significance vary by comorbidity type and national context. In China, the highest odds were for heart disease (OR = 4.93, 95% CI: 2.32–10.51), then joint disease (OR = 4.09, 95% CI: 2.11–7.94) and diabetes (OR = 4.18, 95% CI: 1.90–9.24; Tables 9, 10). Conditional on having positive expenditures, comorbidities meant significantly higher household-level mean OOPHE: US$1,363.24 (CNY 8,897.87; heart disease), US$1,363.74 (CNY 8,901.13; joint disease), and US$2,297.07 (CNY 14,992.98; diabetes). In Vietnam, only hypertension and diabetes combination remained significantly associated with OOPHE (OR = 3.44, 95% CI: 1.26–7.70), while joint and heart disease associations were not significant. Among those with expenditures, comorbidities meant higher mean OOPHE: US$398.61 (VND 9,253,731.15; diabetes), US$268.27 (VND 6,227,888.05; joint disease), and US$222.17 (VND 5,157,676.55; heart disease). Hypertension-related comorbidities were consistently linked to higher out-of-pocket expenditures, with diabetes showing the strongest effect.

**Table 9:** Association of comorbidities with total out of pocket health expenditure (US $) in China.

**Chronic conditions**	**Part I**			**Part II**		
	**Sample size**	**OR (95% CI)**	***P* value**	**Sample size**	**Conditional IE**	***P* value**
Individuals with only hypertension		Ref.				
Individuals with at least hypertension and joint disease	447	4.32 (2.23, 8.38)	< 0.001	226	1,359.13 (685.17, 2,033.08)	< 0.001
Individuals with at least hypertension and heart disease	214	4.93 (2.32, 10.51)	< 0.001	106	1,363.24 (764.73, 1,961.75)	< 0.001
Individuals with at least hypertension and diabetes	192	4.18 (1.90, 9.24)	< 0.001	86	2,297.07 (137.97, 4,456.16)	0.001

All models were conducted separately for groups of identified comorbidities to compare older individuals who have these conditions against older individuals with hypertension only. The identified groups of comorbidities are (1) hypertension and joint, (2) hypertension and heart, and (3) hypertension and diabetes. Part 1 is a logistic regression to predict the probability of individuals incurring any out-of-pocket health expenditure. Part 2 is a generalized linear model with gamma distribution and log link, used to estimate the expected health expenditure given that the health expenditure for order individual is positive.

All models were adjusted for age, sex, household wealth quintile (using household income per capita), household size (number of people in the household), health insurance status.

OR, odds ratio; Conditional IE, conditional incremental effect.

**Table 10:** Association of comorbidities with total out of pocket health expenditure (US $) in Vietnam (*individual level sampling weight applied*).

**Chronic conditions**	**Part I**			**Part II**		
	**Sample size**	**OR (95% CI)**	***P* value**	**Sample size**	**Conditional IE**	***P* value**
Individuals with only hypertension		Ref.				
Individuals with at least hypertension and joint disease	442	2.47 (1.40, 4.37)	0.002	322	268.27 (106.18, 430.35)	0.001
Individuals with at least hypertension and heart disease	356	2.10 (1.07, 4.14)	0.031	251	222.17 (22.26, 422.08)	0.029
Individuals with at least hypertension and diabetes	320	4.44 (2.26, 8.70)	< 0.001	229	398.61 (184.02, 613.20)	< 0.001

All models were conducted separately for groups of identified comorbidities to compare older individuals who have these conditions against older individuals with hypertension only. The identified groups of comorbidities are (1) hypertension and joint, (2) hypertension and heart, and (3) hypertension and diabetes. Part 1 is a logistic regression to predict the probability of individuals incurring any out-of-pocket health expenditure. Part 2 is a generalized linear model with gamma distribution and log link, used to estimate the expected health expenditure given that the health expenditure for order individual is positive.

All models were adjusted for age, sex, household wealth quintile (using an asset-based wealth index), household size (number of people in the household), health insurance status.

OR, odds ratio; Conditional IE, conditional incremental effect.

## Discussion

To our knowledge, this is the first cross-country comparison of multimorbidity-specific financial risk among hypertensive older adults in middle-income settings. The findings show that “multimorbidity” is not a single economic problem: joint, heart, and diabetic clusters impose distinct burdens, and those burdens can be potentially shaped by national financing arrangements. This underscores the need for targeted health financing policies, such as expanding insurance coverage and providing financial protection for high-risk groups, to mitigate the economic impact of multimorbidity. Policymakers should prioritize interventions that address the specific cost drivers of each comorbidity cluster. This finding aligns with the study's objective and corroborates prior evidence from low- and middle-income countries that multimorbidity exacerbates OOP costs and CHE, imposing significant financial strain on households.

Hypertension is the dominant NCD among older adults in both China and Viet Nam, and it seldom occurs alone. In our two provincially representative samples, roughly a quarter of hypertensive older people in China and one-sixth in Viet Nam also reported joint disease; heart-disease co-occurrence was 16.2 vs 10.6%, and diabetes about 10.5 vs 8.5%, respectively. These patterns of comorbidity were accompanied by notably different levels of financial burden in the two countries. Among households with older adults living with hypertension, the incidence of CHE was about four times higher in China (22.6%) than in Viet Nam (6.3%). The OOPHE pattern was consistent across both countries, with the highest costs observed in households with hypertension and diabetes, followed by those with heart disease, and then joint disease.

Financial risk profiles varied by comorbidity cluster and by country. In China, hypertension paired with heart disease was associated with the highest odds of CHE (OR ≈ 3.9) and the probability of incurring OOPHE (OR ≈ 4.9), followed by joint disease and diabetes. In Viet Nam the pattern reversed: diabetes combinations generated the greatest financial pressure (CHE OR ≈ 9.4; OOPHE OR ≈ 4.4), while heart-disease clusters were not significantly different from hypertension alone. Service-specific spending patterns differed in magnitude but were similar in composition: outpatient care accounted for the largest share of expenditures among households in both China and Viet Nam, though inpatient costs remained substantial for some households.

Our analysis reveals that among older adults with hypertension, the presence of heart disease in China and diabetes in Viet Nam is associated with increased financial burdens. In China, among hypertensive older adults, coexisting heart disease raised the likelihood of any OOPHE by 4.9-fold and of CHE by 3.9-fold relative to hypertension alone. The finding is consistent with the high prevalence of cardiovascular disease in China, where rates nearly doubled between 1990 and 2019 (19). The management of CVD often involves complex and costly treatments, including hospital admissions and procedural interventions, which may contribute to the higher financial burden observed (20, 21). In Viet Nam, having an older person with the coexistence of hypertension and diabetes confers the largest incremental financial burden among the multimorbidity clusters pushing household CHE odds up nine-fold. Managing diabetes requires ongoing expenses for medications, monitoring supplies, and regular healthcare visits. The evidence indicating that older patients with diabetes in Vietnam bearded substantial costs that attributed to diabetes-related complications and associated hospitalization due to poor management of diabetes (22, 23). The financial strain is further exacerbated for older adults living alone, a demographic more prevalent among Vietnamese participants in our study (24). The lack of familial support may limit their ability to manage chronic conditions effectively, leading to increased healthcare utilization and associated costs (25).

The stark difference in financial outcomes between two countries are found in the current study may reflect the discrepancies in policy orientation toward UHC and service delivery models. First, the China's higher CHE incidence despite similar health insurance coverage compared to Vietnam suggests that China's health insurance offers shallower financial protection. Indeed, China rapidly achieved broad population coverage but covers a limited range of services, leaving patients to pay a large share of costs out-of-pocket (26, 27). In China, most older adults are enrolled in one of three social health insurance schemes, including Urban Employee Basic Medical Insurance (UEBMI), Urban Resident Basic Medical Insurance (URBMI), and the New Rural Cooperative Medical Scheme (NCMS) (28). The service coverage is based on the level of funding in different schemes, for example, the scheme with the highest financial support (UEBMI) has the best service coverage and highest reimbursement rates while health service included in NCMS and URBMI in China is limited due to a low level of funding, resulting in significant variance in service coverage. Vietnam, on the other hand, expanded insurance more gradually with a relatively comprehensive benefits package for the poor and older adults, resulting in better protection against catastrophic spending (13). Service coverage is the identical for every Vietnamese enrolee regardless of the source of financial contributions, either state subsidized groups or voluntary group. This systemic difference likely explains why Vietnamese older adults were less prone to CHE, their insurance more effectively covers essential services, whereas Chinese patients often still face high uncovered expenses (13). In our analysis, nearly all older hypertensive adults had insurance coverage in both countries; hence, the gap in CHE reflects differences in coverage depth and health care costs, rather than enrolment.

Our findings reveal distinct patterns in the association between comorbidity clusters and CHE across China and Viet Nam. In China, heart disease conferred the highest CHE risk among hypertensive individuals (OR = 4.32), followed by joint disease and diabetes, while in Viet Nam, diabetes was the dominant driver (OR = 9.35), with heart disease showing no significant association. Similar patterns OOPHE, with all three clusters significantly associated with OOPHE in China, but only the hypertension–diabetes cluster in Viet Nam. These differences likely reflect broader health system and social context: China's hospital-centric, high-cost cardiovascular care model and limited outpatient coverage amplify financial risk, especially for heart disease, whereas Viet Nam's primary care system contains hypertension-related costs but offers limited protection for diabetes care.

The same combinations of hypertension and comorbid conditions generate different financial burdens in China and Viet Nam can be partially explained by key differences in health system design. This contrast reflects China's hospital-centric service model, where older patients frequently bypass primary care, leading to high-cost cardiac interventions and substantial out-of-pocket payments, even for insured individuals (11). Although China's National Essential Public Health Service Package has improved hypertension control, most cardiovascular referrals still occur in urban hospitals (29). The resulting uncovered copayments possibly explain the heart-disease cluster's high CHE odds. For instance, the average direct medical costs for ischaemic heart disease can exceed CNY 27,000 (≈US$4,300) per admission among insured patients enrolled in UEBMI scheme (30). Another study also found that older patients with CVD to pay more out of pocket, with the spending highly concentrated in a small number of individuals; however, this group benefits less from medical insurance reimbursements, meaning insurance covers less of their total costs compared to younger patients (21). Additionally, the relatively lower CHE risk observed among older people with hypertension–diabetes combinations may also reflect China's recent policy efforts to improve outpatient care coverage. Since 2019, the Chinese government has progressively expanded outpatient reimbursement for essential chronic disease management, including coverage for drugs listed in the National Reimbursement Drug List delivered through outpatient settings (29). A 2021 national guideline further called for pooling outpatient costs for common and chronic conditions like hypertension and diabetes under the basic medical insurance system (29). These reforms mark a shift toward strengthening financial protection for high-frequency outpatient care, which may partly explain why the diabetes cluster in China is associated with lower odds of CHE than heart-disease clusters, despite the overall high burden of diabetes.

Viet Nam's primary care system plays a central role in managing routine hypertension, with grassroots facilities such as commune health stations (CHSs) and district health centers delivering most first-contact care (31, 32). CHSs now manage up to 80% of mild-to-moderate NCDs using standardized protocols, which helps contain costs and contributes to relatively low OOP spending for hypertension management (32). However, diabetes care faces substantial gaps at the primary care level, highlighting significant unmet needs. Essential diabetes management services—including HbA1c testing, insulin provision, and glucose monitoring—are often unavailable, forcing patients to seek routine monitoring and complication treatment at district or provincial hospitals. Copayments at these higher-level facilities typically range from 5 to 20%, substantially increasing the financial burden. One national study found that 55% of patients with diabetes experienced complications, and that approximately 70% of direct medical costs were attributable to complication-related treatment (23). Furthermore, the total cost of care for patients with complications was nearly double that of those without. Importantly, non-medical costs (e.g., transportation, caregiving) and indirect costs (e.g., productivity loss) accounted for an additional 12 and 24% of total costs, respectively, expenses that are almost entirely paid out-of-pocket (23). These findings suggest that, despite Viet Nam's strong primary care infrastructure, households with older adults suffering from both hypertension and diabetes still face disproportionately high financial risks. Our findings have several implications for strengthening financial protection and chronic disease management in aging populations across China and Viet Nam. First, given the significant out-of-pocket burden associated with multimorbidity, particularly for heart disease in China and diabetes in Viet Nam, targeted reforms in health financing are warranted. In China, this may include capping inpatient co-payments for high-cost cardiovascular admissions and accelerating efforts to shift care toward a primary care-led system through realigned provider incentives and accountability mechanisms. The government's promotion of two-way referral systems and integrated care pathways offers a platform for such change (33). In Viet Nam, strengthening the capacity, ensuring sufficient resources, such as, medications (both oral medication and insulin) and medical supplies, and service availability, especially hypertension and diabetes management (glucose monitoring and basic para-clinical tests for complication screening) at commune health station, would help reduce recurrent household spending due to avoidable service utilization at higher-level health facilities. Second, both countries could benefit from bundling common multimorbidity clusters (e.g., hypertension–diabetes or hypertension–heart disease) into essential service packages and linking insurance reimbursement more directly with early detection and integrated community-based care. These reforms would not only mitigate avoidable complications but also enhance the efficiency of resource allocation in the context of population aging and rising NCD prevalence.

This study offers valuable comparative insights using large, provincially representative datasets and harmonized analytic strategies across China and Viet Nam. Adjustments for household wealth, demographic factors, and disease profiles strengthen the internal validity of observed associations between comorbidity clusters and financial risk. However, several limitations should be acknowledged. First, the cross-sectional nature of the data restricts causal inference, and the findings should be interpreted as associations rather than impacts. *Second, it should be noted that the data items collected are not perfectly harmonized between the CHARLS and Viet Nam datasets. However, this limitation does not impede the central analysis, as our primary focus is on assessing the differential impact of hypertensive morbidities on health expenditures in China vs. Viet Nam*. Third, the identification of chronic conditions in the Viet Nam sample relies on self-reported diagnoses, which may be subject to under-reporting or recall bias. In contrast, hypertension in China was determined using both blood pressure measurements and treatment history. This discrepancy in disease ascertainment between the two countries could introduce bias, particularly for under-diagnosed conditions such as diabetes, and should be considered when interpreting cross-country comparisons. Similarly, health expenditure data may be affected by reporting inaccuracies in both countries. Lastly, while cost estimates were converted into US dollars, the absence of purchasing power parity adjustments may limit the precision of cross-country cost comparisons, as differences in local price levels and cost of living could affect the relative magnitude of financial burden between China and Viet Nam. Furthermore, future research could enhance policy relevance by estimating average marginal effects (AMEs) to capture the overall economic burden and exploring its heterogeneity across key socioeconomic subgroups. Despite these limitations, the study provides timely and policy-relevant evidence on the economic burden of multimorbidity among older adults in two rapidly aging health systems.

## Conclusions

Multimorbidity significantly amplifies the financial burden faced by older adults with hypertension, but the extent and pattern of this burden vary by comorbidity type and health system context. Our comparative analysis highlights how heart disease in China and diabetes in Viet Nam each pose distinct risks of out-of-pocket spending and catastrophic health expenditure, shaped by differences in service delivery models and universal insurance coverage strategies. These findings call for immediate policy action in middle-income Asian countries to protect households from financial hardship. Policymakers should expand outpatient coverage for chronic disease care, cap high-cost inpatient co-payments, and incorporate common multimorbidity clusters into essential service packages. In parallel, investing in primary care-led, community-based integrated care models will enable early detection, coordinated management, and lower long-term costs. Achieving healthy aging in the region will require policies that are not only fiscally responsive but also aligned with the complex needs of older adults living with multiple chronic conditions.

Building on this work, future research should explore longitudinal trajectories of multimorbidity and financial risk using upcoming waves of CHARLS and prospective Vietnamese panel data to better understand causality. Longitudinal data are particularly valuable for policymaking because they can identify temporal patterns, and the timing of financial strain, allowing policymakers to design targeted interventions and allocate resources more effectively. Further studies should also examine other prevalent comorbidities, and assess intangible costs, such as productivity loss and caregiver burden, that were beyond the scope of this study but are critical to capturing the full societal impact of chronic disease in aging populations.

## Data Availability

Publicly available datasets were analyzed in this study. This data can be found here: CHARLS: https://charls.pku.edu.cn/. VietNam data: the data supporting this study are restricted but may be available from the authors upon reasonable request and with permission from the Health Strategy and Policy Institute.
