# Correction: Economic burdens of health expenditure for multi-morbidity of older people with hypertension in China and Vietnam

**DOI:** 10.3389/fpubh.2025.1721839

**Published:** 2025-10-27

**Authors:** Nguyen Khanh Phuong, Nguyen Hoang Giang, Haolin Li, Nguyen The Vinh, Tran Thi Mai Oanh, Chenkai Wu

**Affiliations:** ^1^Health Strategy and Policy Institute, Hanoi, Vietnam; ^2^Global Health Research Center, Duke Kunshan University, Kunshan, China

**Keywords:** hypertension, multimorbidity, financial burden, older adults, low- and middle-income countries

An incorrect **Funding** statement was provided. The correct funder is “Kunshan Municipal Government.”

An incorrect number was provided for “Kunshan Municipal Government.” The correct number is [24KKSGR016].

The correct **Funding** statement reads:

“The author(s) declare that financial support was received for the research and/or publication of this article. The research was supported by the Kunshan Municipal Government Research Funding (24KKSGR016).”

The original version of this article has been updated.

