# Supplementary material for: Economic burdens of health expenditure for multi-morbidity of older people with hypertension in China and Vietnam
Source: Front Public Health. 2025 Sep 26;13:1666119. doi: 10.3389/fpubh.2025.1666119 (PMC12510917; doi:10.3389/fpubh.2025.1666119)
Supplement: Supplementary file 1 [file Table_1.docx]

**Appendix table 1.**

|  | **China** | | | | **Viet Nam** | | | |
| --- | --- | --- | --- | --- | --- | --- | --- | --- |
|  | **Sichuan** | **Jiangsu** | **Yunnan** | **Total** | **Thanh Hoa** | **Tien Giang** | **Yen Bai** | **Total** |
| **Demo-socioeconomic background of the provinces** | | | | | | | | |
| Geography | Inland province with mountains and plateaus | Eastern coastal province with plains | Southwestern mountainous province |  |  |  |  |  |
| Total provincial population | 83,400,000 | 80,500,000 | 48,300,000 |  |  |  |  |  |
| % urban population | 31.2 | 65.5 | 33.7 |  |  |  |  |  |
| GDP per capita (USD) | 7,387 | 17,445 | 5,629 |  |  |  |  |  |
| **Socioeconomic background of the survey sample** | | | | | | | | |
| Number of participants | 603 | 357 | 398 | 1,358 | 512 | 512 | 512 | 1,536 |
| Sex, n (%) |  |  |  |  |  |  |  |  |
| Male | 297 (49.2%) | 173 (48.5%) | 200 (50.3%) | 670 (49.3%) | 224 (43.8%) | 188 (36.7%) | 220 (43.0%) | 632 (41.1%) |
| Female | 306 (50.8%) | 184 (51.5%) | 198 (49.7%) | 688 (50.7%) | 288 (56.3%) | 24 (63.3%) | 292 (57.0%) | 904 (58.9%) |
| Age, mean (SD) | 69.96 (7.00) | 69.48 (7.12) | 69.17 (7.71) | 69.60 (7.25) | 69.8 (8.22) | 69.89 (7.28) | 71.94 (7.95) | 70.54 (7.88) |
| Age groups, n (%) |  |  |  |  |  |  |  |  |
| 60-69 | 324 (53.7%) | 205 (57.4%) | 241 (60.6%) | 770 (56.7%) | 303 (59.2%) | 283 (55.3%) | 206 (40.4%) | 792 (51.6%) |
| 70-79 | 209 (34.7%) | 115 (32.2%) | 112 (28.1%) | 436 (32.1%) | 134 (26.2%) | 169 (33.0%) | 212 (41.6%) | 515 (33.6%) |
| 80+ | 70 (11.6%) | 37 (10.4%) | 45 (11.3%) | 152 (11.2%) | 75 (14.6%) | 60 (11.7%) | 92 (18.0%) | 227 (14.8%) |
| Marital Status, n (%) | 460 (76.3%) | 286 (80.1%) | 292 (73.4%) | 1,038 (76.4%) | 358 (69.9%) | 256 (50.0%) | 312 (60.9%) | 926 (60.3%) |
| Place of residence, n (%) |  |  |  |  |  |  |  |  |
| Urban | 188 (31.2%) | 218 (61.1%) | 148 (37.2%) | 554 (40.8%) | 256 (50.0%) | 256 (50.0%) | 256 (50.0%) | 768 (50.0%) |
| Rural | 415 (68.8%) | 139 (38.9%) | 250 (62.8%) | 804 (59.2%) | 256 (50.0%) | 256 (50.0%) | 256 (50.0%) | 768 (50.0%) |
| Education level, n (%) |  |  |  |  |  |  |  |  |
| Illiterate | 203 (33.6%) | 142 (39.7%) | 171 (43.0%) | 516 (38.0%) | 18 (3.5%) | 25 (4.9%) | 8 (1.6%) | 51 (3.3%) |
| Below elementary school | 165 (27.4%) | 57 (16.0%) | 95 (23.9%) | 317 (23.3%) | 69 (13.5%) | 209 (40.8%) | 27 (5.3%) | 305 (19.9%) |
| Elementary school | 165 (27.4%) | 75 (21.0%) | 89 (22.4%) | 329 (24.2%) | 83 (16.2%) | 128 (25.0%) | 93 (18.2%) | 304 (19.8%) |
| Middle school and above | 70 (11.6%) | 83 (23.3%) | 43 (10.8%) | 196 (14.4%) | 341 (66.7%) | 150 (29.3%) | 384 (75.0%) | 875 (57.0%) |
| Household income per capita |  |  |  |  |  |  |  |  |
| Below $1,900 | 184 (30.5%) | 20 (5.6%) | 134 (33.7%) | 338 (24.9%) | na | na | na | na |
| $1,900 - $5,707.5 | 155 (25.7%) | 79 (22.1%) | 105 (26.4%) | 339 (25.0%) | na | na | na | na |
| $5,707.5 - $15,930 | 144 (23.9%) | 99 (27.7%) | 94 (23.6%) | 337 (24.8%) | na | na | na | na |
| $15,930 above | 120 (19.9%) | 159 (44.4%) | 65 (16.3%) | 344 (25.3%) | na | na | na | na |
| Household wealth quintile (using asset-based wealth index) |  |  |  |  |  |  |  |  |
| Q1 (Poorest) | 143 (23.7%) | 13 (3.6%) | 116 (29.1%) | 272 (20.0%) | 91 (17.8%) | 118 (23.0%) | 89 (17.4%) | 298 (19.4%) |
| Q2 | 146 (24.2%) | 48 (13.5%) | 78 (19.6%) | 272 (20.0%) | 92 (18.0%) | 138 (27.0%) | 80 (15.6%) | 310 (20.2%) |
| Q3 | 109 (18.1%) | 69 (19.3%) | 93 (23.4%) | 271 (20.0%) | 64 (12.5%) | 155 (30.3%) | 95 (18.6%) | 314 (20.4%) |
| Q4 | 117 (19.4%) | 98 (27.5%) | 58 (14.6%) | 273 (20.1%) | 103 (20.1%) | 81 (15.8%) | 125 (24.4%) | 309 (20.1%) |
| Q5 (Richest) | 88 (14.6%) | 129 (36.1%) | 53 (13.3%) | 270 (19.9%) | 162 (31.6%) | 20 (3.9%) | 123 (24.0%) | 305 (19.9%) |
| Insurance coverage, n (%) |  |  |  |  |  |  |  |  |
| Yes | 580 (96.2%) | 354 (99.2%) | 387 (97.2%) | 1,321 (97.3%) | 488 (95.3%) | 481 (93.9%) | 512 (100.0%) | 1,481 (96.4%) |
| No | 23 (3.8%) | 3 (0.8%) | 11 (2.8%) | 37 (2.7%) | 24 (4.7%) | 31 (6.1%) | 0 (0.0%) | 55 (3.6%) |
| Smoking status, n (%) |  |  |  |  |  |  |  |  |
| Never smoked | 349 (57.9%) | 200 (56.0%) | 197 (49.5%) | 746 (54.9%) | na | na | na | na |
| Previous smoked | 105 (17.4%) | 64 (17.9%) | 69 (17.3%) | 238 (17.5%) | na | na | na | na |
| Current smoking | 149 (24.7%) | 93 (26.1%) | 132 (33.2%) | 374 (27.5%) | na | na | na | na |

**Appendix table 2.** Prevalence of two chronic conditions with a prevalence greater than 2% of each total participants*

| **Comorbidities** | **China, n (%)**  **N=10,800** | **Vietnam, n (%)**  **N=1,535** |
| --- | --- | --- |
| **Hypertension + Joint disease** | **2,428 (22.5%)** | **247 (16.1%)** |
| **Hypertension + Heart disease** | **1,752 (16.2%)** | **161 (10.5%)** |
| **Hypertension + Diabetes** | **1,144 (10.6%)** | **131 (8.5%)** |
| Hypertension + Digestive disease | 1,613 (14.9%) |  |
| Hypertension + Dyslipidemia | 1,812 (16.8%) |  |
| Hypertension + Chronic lung disease | 1,103 (10.2%) |  |
| Hypertension + Stroke | 802 (7.4%) |  |
| Hypertension + Kidney disease | 709 (6.6%) |  |
|  |  |  |
| **Heart disease + Joint disease** | **1,428 (13.2%)** | **87 (5.7%)** |
| Heart disease + Digestive disease | 1,179 (10.9%) |  |
| Heart disease + Dyslipidemia | 1,168 (10.8%) |  |
| Heart disease + Chronic lung disease | 824 (7.6%) |  |
| Heart disease + Diabetes | 655 (6.1%) |  |
| Heart disease + Kidney disease | 548 (5.1%) |  |
|  |  |  |
| Diabetes + Dyslipidemia | 876 (8.1%) |  |
| Diabetes + Joint disease | 799 (7.4%) |  |
| Diabetes + Digestive disease | 546 (5.1%) |  |
|  |  |  |
| Digestive disease + Joint disease | 2,056 (19.0%) |  |
| Digestive disease + Dyslipidemia | 1,015 (9.4%) |  |
| Digestive disease + Chronic lung disease | 952 (8.8%) |  |
| Digestive disease + Stroke | 360 (3.3%) |  |
| Digestive disease + Kidney disease | 618 (5.7%) |  |
|  |  |  |
| Stroke + Joint disease | 542 (5.0%) |  |
|  |  |  |
| Joint disease + Dyslipidemia | 1,320 (12.2%) |  |
| Joint disease + Chronic lung disease | 1,204 (11.1%) |  |
| Joint disease + Kidney disease | 747 (6.9%) |  |
|  |  |  |
| Dyslipidemia + Chronic lung disease | 623 (5.6%) |  |

*Only combinations with higher than 5% prevalence were presented
